# Ecological Mercenaries: Why Aphids Remain Premier Models for the Study of Ecological Symbiosis

**DOI:** 10.3390/insects16101000

**Published:** 2025-09-25

**Authors:** Roy A. Kucuk, Benjamin R. Trendle, Kenedie C. Jones, Alina Makarenko, Vilas Patel, Kerry M. Oliver

**Affiliations:** Department of Entomology, University of Georgia, Athens, GA 30602, USA; benjamin.trendle@uga.edu (B.R.T.); kenedie.conaway@uga.edu (K.C.J.); alina.makarenko@uga.edu (A.M.); patelva@uga.edu (V.P.)

**Keywords:** facultative symbionts, defensive mutualism, host–parasitoid, host–microbe, *Hamiltonella defensa*, insect pathogens, climate change, biological control, coevolution, heritable symbionts

## Abstract

Aphids remain exceptional models for symbiosis research due to their unique experimental advantages that extend beyond documenting symbiont-mediated phenotypes. Nine commonly occurring facultative bacterial symbionts provide well-characterized benefits, including defense against parasitoids, pathogens, and thermal stress. Yet the system’s greatest value lies in enabling diverse research applications across biological disciplines through experimental tractability combined with ecological realism. Researchers can create controlled experimental lines through symbiont manipulation, maintain clonal host populations indefinitely, and cultivate symbionts independently. This experimental power is complemented by extensive knowledge of symbiont dynamics in natural populations, including temporal and geographic distribution patterns—features generally unavailable in other insect-microbe systems. These advantages facilitate investigation of key processes in symbiosis, including transmission dynamics, mechanisms, strain-level functional diversity, multi-partner infections, and transitions from facultative to co-obligate relationships. Integration across biological scales—from genomics to field ecology—enables research on symbiont community assembly, ecological networks, coevolutionary arms races, and agricultural applications. This combination of experimental flexibility, comprehensive natural history knowledge, and applied relevance positions aphids as invaluable for advancing symbiosis theory while addressing practical challenges in agriculture and invasion biology.

## Simple Summary

Aphids are plant-feeding insects that rank among the world’s most significant agricultural pests, transmitting viruses and causing billions of dollars in crop losses annually. They have also emerged as premier model organisms for studying microbial symbiosis. Most aphids harbor facultative bacterial symbionts that provide conditional benefits, including protection against parasitoid wasps, pathogenic fungi and viruses, and thermal stress. The aphid–symbiont system offers researchers unique experimental advantages: symbionts can be transferred between host lines, selectively eliminated, and cultured independently, allowing precise isolation of symbiont effects on aphid biology. Simultaneously, extensive field studies have documented symbiont prevalence, seasonal dynamics, and ecological impacts in natural populations—a depth of knowledge rarely available in other host-microbe systems. This integration of experimental tractability with ecological realism enables investigation of fundamental evolutionary questions, including how defensive partnerships evolve, how they spread through populations, and their cascading effects on ecological networks. Beyond basic research, these studies inform practical applications in biological control and pest management. Symbiont-mediated resistance can compromise the effectiveness of parasitoid biocontrol, yet it also presents opportunities for novel management strategies that leverage protective symbionts to control pathogenic virus transmission.

## 1. Introduction

Symbiotic relationships have fundamentally shaped the evolution of multicellular eukaryotes, with insects providing some of the most striking examples [1]. Most insect species maintain intimate microbial associations that enhance nutrient acquisition or host defense [2]. These symbioses manifest in two general ways: a subset of environmental microbes takes up residence in exposed tissues, including the digestive tract where they function to break down recalcitrant plant polymers or protect against ingested pathogens [3,4]; and heritable symbionts, passed faithfully from mother to offspring, conferring ecological benefits or acting as reproductive parasites [5]. Obligate heritable symbionts repeatedly enabled insect groups to colonize and radiate in nutritionally challenging niches, such as vertebrate blood or plant sap [6,7], while facultative heritable symbionts provide host defense or mediate other ecological interactions [8]. Aphids (Aphididae: Hemiptera) serve as valuable models, particularly for studying function in facultative symbionts. This review highlights aphids’ continued significance for investigating symbiont-conferred phenotypes, providing a brief historical perspective while noting the system’s advantages and limitations. We demonstrate how the aphid model has advanced understanding of facultative symbiosis while also serving as a powerful system for investigating fundamental questions in ecology and evolution, then identify research directions that capitalize on both applications.

## 2. Discovery of Aphid Facultative Symbionts

**Figure 1:**
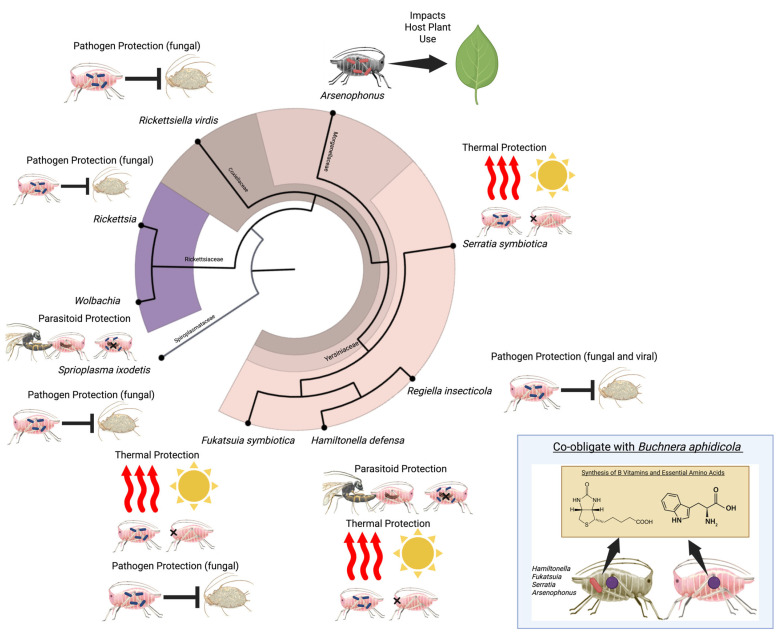
Summary of phenotypes conferred to aphids by their common facultative symbionts. Symbionts provide varied services, including mediating dietary breadth, and providing anti-parasitoid defense, anti-fungal defense, anti-viral defense, thermal protection, and nutritional assistance. Experimentally documented phenotypes are organized along the bacterial phylogeny, with pathogen and parasitoid species listed alongside the aphid species in which phenotypes were observed. Created in BioRender. Trendle, B.R.; Kucuk, R.A. (2025). https://BioRender.com/0h1tml7.

Aphids are phloem-feeding insects that vector plant pathogenic viruses, making many serious crop pests [9,10]. Phloem lacks essential nutrients, and most aphid species rely on *Buchnera aphidicola* for nutritional supplementation [11]. This obligate symbiont, required by all individual aphids, inhabits specialized cells called bacteriocytes, which form an organ called the bacteriome. Paul Buchner and colleagues extensively documented aphid bacteriomes through light microscopy [12], but the advent of modern molecular methods [13] was required to formally identify *Buchnera* (Enterobacterales) [14]. These microscopy studies revealed that some individual aphids carried additional bacteria referred to as facultative or secondary symbionts [12]. *Serratia symbiotica* was the first facultative symbiont identified through sequencing [15] and eight additional widely occurring symbionts have since been recognized [16,17]: *Spiroplasma*, *Regiella insecticola*, *Hamiltonella defensa*, *Rickettsia*, *Arsenophonus*, *Wolbachia*, *Fukatsuia symbiotica*, and *Rickettsiella viridis* (Figure 1) [18,19,20,21,22,23,24,25,26,27]. Additional symbiont lineages have been reported in aphids [16,19,28,29,30], but these require further study to elucidate functional roles and determine whether they are broadly distributed across aphids.

## 3. Aphid Facultative Symbionts Mediate Diverse Interactions

### 3.1. Experimental Foundations and Methodological Advances

Early studies identified correlations between aphid facultative symbionts and ecological traits [31,32], leading to the development of two key experimental approaches for isolating symbiont roles: microinjection of symbiont-containing hemolymph from donor to recipient aphids [33] and selective elimination of facultative symbionts while preserving *Buchnera* [34]. Aphids’ cyclical parthenogenesis enables indefinite maintenance of clonal lines, which, combined with symbiont manipulation, allows for precise isolation of symbiont-conferred phenotypes [16]. Initial experimental studies revealed that *Serratia* and *Rickettsia* conferred heat tolerance [35,36], *Hamiltonella* protected against parasitoid wasps [37], and *Regiella* provided resistance to fungal entomopathogens [38]. In total, facultative symbionts dramatically shape aphid ecology and evolution through diverse phenotypic effects (Figure 1).

### 3.2. Defensive Services Against Natural Enemies

Collectively, the nine common aphid facultative symbionts enhance thermal tolerance and defend against natural enemies, with most influencing multiple ecological interactions (Figure 1).

Defense against parasitoids remains the most thoroughly studied symbiont-mediated phenotype. *Hamiltonella* [39,40], *Spiroplasma* [41], and certain *Regiella* strains [42] protect against parasitoid wasps, while *Serratia* can reduce parasitoid success without conferring direct benefits to aphids [37,43]. This protection spans multiple aphid species—including pea, black bean, cowpea, and bird-cherry oat aphids—against at least six parasitoid species from braconid (Ichneumonoidea) and aphelinid (Chalcidoidea) wasps [37,44,45,46].

Protection against fungal pathogens represents the most widespread defensive trait, documented in six symbionts: *Regiella*, *Rickettsia*, *Rickettsiella*, *Spiroplasma*, *Fukatsuia*, and *Wolbachia* [47,48,49,50]. This defense proves effective against specialized fungal pathogens but not generalists like *Beauveria bassiana* [50,51]. Less is known about protection against other pathogen types, though *Regiella* was recently found to protect against aphid-specific viruses, e.g., *A. pisum* virus (APV) [52], suggesting broader antimicrobial capabilities that align with *Wolbachia*’s protective effects against diverse pathogen groups in other host systems [53].

### 3.3. Plant Interactions and Dietary Specialization

Facultative symbionts influence aphid–plant interactions through multiple pathways. Studies across pea aphid biotypes revealed correlations between food plants and specific symbionts, including *Regiella* enrichment in clover-feeding populations [20,31,32,54,55]. While early experiments showed *Regiella* enhanced performance on white clover [55], subsequent studies revealed outcomes depend on complex interactions among aphid genotypes, symbiont strains, and plant species [56,57]. The clearest demonstration of symbiont-mediated dietary breadth comes from cowpea aphids (*Aphis craccivora*), where *Arsenophonus* improved performance on locusts while reducing fitness on alfalfa [58].

Symbionts colonizing salivary glands and stylets can modify plant signaling and chemistry, potentially improving aphid fitness and affecting plant pathogen transmission [59,60,61,62]. For example, *Hamiltonella*-infected aphids may trigger reduced plant volatile emissions, lowering parasitoid attraction and attack rates [63]—demonstrating dual protection through direct wasp killing and indirect attack reduction. Further research is needed on symbiont-mediated food plant specialization, underlying mechanisms, and implications for aphid pest status.

### 3.4. Thermal Tolerance and Climate Adaptation

Geographic distribution and ecological niche expansion depend partly on thermal tolerance, which may be constrained by temperature-sensitive symbionts [64]. The obligate symbiont *Buchnera*, having undergone extensive genome reduction, is heat-sensitive and likely constrains aphid distributions [65]. Many facultative symbionts—*Serratia*, *Hamiltonella*, *Regiella*, *Fukatsuia*, and *Rickettsia*—enhance aphid survival after heat shocks -short bursts of very high heat, possibly by supporting *Buchnera* recovery [35,48,66,67,68]. *Serratia* may also prevent recurrent mutations in *Buchnera*’s heat shock protein gene *ibpA*, crucial for bacteriocyte stabilization under heat stress [69].

The diversity of thermal-tolerance-conferring symbionts and *Serratia*’s persistent protective effects through multiple heat exposures [70] may provide resilience in a warming world. However, while numerous symbionts help aphids survive brief temperature spikes, their role under sustained climate stressors, including heat waves and increased mean temperatures, remains unclear. Additionally, symbiont-mediated traits often deteriorate under warming—*Hamiltonella*’s anti-parasitoid benefits decline significantly at moderately elevated temperatures [71,72]. However, given that symbionts are often a source of rapid adaptation, thermally robust defensive strains may emerge and spread as temperatures rise.

### 3.5. Conditional Benefits Are Often Paired with Costs

While providing diverse benefits, facultative symbionts impose significant costs manifesting as constitutive costs (direct metabolic costs of symbiont maintenance) and induced costs during enemy encounters (e.g., when defensive toxins damage aphid tissues) [73]. Cost magnitude and nature vary with aphid and symbiont genotypes, often correlating with protective benefits, though not invariably [74,75,76,77]. Population cage experiments provide compelling cost evidence, as protected aphid lines show reduced fecundity compared to symbiont-free controls in direct competition [78].

Cost–benefit relationships become more complex when considering multiple threats. While some symbionts protect against multiple challenges [48], others defend against one threat while increasing vulnerability to another. These conditional costs have been documented for insecticides ([79], but see [80]), predators [81], and viral pathogens [52]. Conversely, in enemy-free environments, particular symbionts including *Wolbachia* and *Arsenophonus* enhance aphid fitness through mechanisms that remain uncharacterized [50,82].

### 3.6. Nutritional Supplementation and Co-Obligate Evolution

While obligate symbionts initially enable evolutionary innovation and niche colonization, long-term host restriction and transmission bottlenecks create vulnerabilities requiring compensation [83]. In aphids, obligate symbiont rescue has repeatedly occurred through co-obligate symbiont acquisition, often from the facultative symbiont pool, providing B vitamins (riboflavin and biotin) or amino acids (histidine and tryptophan) that *Buchnera* can no longer supply [84,85,86,87,88]. Co-obligate symbiosis has evolved independently at least six times, involving ten facultative symbiont species, and potentially occurs in more than 10% of aphid species [27]. Beyond co-obligate relationships, facultative symbionts may affect aphid nutrition by influencing *Buchnera* abundance on low-nitrogen diets [23,89].

### 3.7. Future Directions and Research Priorities

The taxonomic diversity within aphid–symbiont systems represents a barrier to identifying general principles governing these interactions. Current knowledge is concentrated in a few model systems, leaving most taxa inadequately characterized and creating significant gaps in our understanding of symbiont functional diversity. While facultative symbionts occur across most of the ~5000 aphid species, their phenotypic effects have been studied primarily in pea aphids (*Acyrthosiphon pisum*) and a handful of other agriculturally important species, creating taxonomic and ecological biases. This restricted focus has resulted in uneven coverage across aphid and symbiont lineages, limiting the identification of general principles governing facultative symbiont–aphid interactions. Phenotypic characterization across additional aphid taxa—including those in natural ecosystems—would enable comparative analyses to reveal whether symbiont-mediated traits represent convergent solutions to shared ecological challenges or reflect lineage-specific evolutionary innovations. Such taxonomically broad studies could uncover currently invisible patterns in symbiont function, including whether certain defensive services are more prevalent in particular aphid clades and how ecological niche correlates with symbiont diversity. Recent discoveries of anti-viral protection [39], pathogen transmission modification [40,41], and *Wolbachia’s* first documented role in aphids [42] suggest we have only begun uncovering symbiont-mediated services.

## 4. Transmission and Tissue Tropism

### 4.1. Tissue Distribution and Cellular Mechanisms

Unlike the obligate symbiont *Buchnera*, which is restricted to bacteriocytes, facultative symbionts inhabit multiple tissues including bacteriocytes, sheath cells, and hemolymph [90,91]. Facultative symbiont abundance fluctuates with host life stage [92], genotype [93], and environmental stress [94], reflecting their more flexible associations with hosts. Cellular studies reveal that *Serratia* (and presumably other facultative symbionts) achieves vertical transmission by exploiting the endocytotic machinery that evolved for *Buchnera* transfer, with cells residing in the hemolymph being incorporated into embryos [90].

### 4.2. Vertical and Horizontal Transmission Dynamics

Transmission patterns for facultative symbionts are complex and context-dependent. While these symbionts are primarily vertically transmitted with high maternal transfer rates under controlled laboratory conditions [95], horizontal transmission within and among species also occurs occasionally [16]. Transmission patterns in natural populations remain incompletely understood, although significant declines in symbiont frequencies during host overwintering pea aphids have been observed [96,97]. Symbiont acquisition (or loss) can occur during sexual reproduction [98,99,100] and horizontal transfer can happen via food plants [101] or parasitoid contact [102], with host relatedness impacting rates of lateral transfer [103,104].

Despite constraints, facultative symbionts retain remarkable transfer capabilities that become particularly evident during host colonization of new ecological niches. Their convergent acquisition in similar environments worldwide suggests these microbes function as a shared genetic resource, facilitating rapid host adaptation across diverse habitats and enabling swift acquisition of new ecological traits [16,105].

### 4.3. Evolutionary Insights and Knowledge Gaps

The transitional nature of facultative symbionts—evolving from free-living commensals or pathogens to symbionts, or progressing from facultative to obligate relationships—provides exceptional opportunities to understand symbiosis evolution. For example, *Serratia* reveals potential evolutionary pathways by which environmental pathogens transition to heritable symbionts through comparative studies of transmission mechanisms and genomic differences between symbiotic and pathogenic strains [106]. However, the molecular mechanisms governing tissue colonization, population persistence, and reliable transmission remain largely unexplored for most facultative symbionts [103]. Additionally, rates of vertical transmission failure and of horizontal transmission under natural field conditions are poorly understood, despite their critical importance for infection dynamics in wild populations (see below). Understanding these transmission dynamics would provide critical predictive frameworks for both applied pest management strategies and fundamental microbial ecology. Moreover, facultative symbiont systems offer unique experimental opportunities to elucidate broader principles of host–microbe interactions, including mechanisms of pathogenesis and transmission that extend well beyond arthropod systems to medically relevant microorganisms.

## 5. Facultative Symbionts in Natural Populations

### 5.1. Survey Approaches and Distribution Patterns

Facultative symbionts in natural aphid populations are widespread but unevenly distributed across species and populations. Early field surveys of facultative symbionts typically involved limited aphid and symbiont species [20], but more comprehensive surveys have since been undertaken. These include investigations of specific aphid species: pea aphids [107], cowpea aphids [108], grain aphids [109], *Cinara* aphids [110], and Hormaphidinae [111]. Other studies have focused on specific locations, such as the Netherlands [112] or target specific symbionts like *Wolbachia* [113,114].

### 5.2. Temporal Dynamics and Ecological Drivers

Field studies collectively reveal dramatic variation in facultative symbiont infection frequencies (0–100%) among aphid populations, and within the same aphid species. Symbiont frequencies can change rapidly in response to enemy pressure [96,115] or other environmental factors [97,116], demonstrating the dynamic nature of these associations in natural settings. Paradoxically, while facultative symbionts provide documented protection under field conditions, they do not always increase in abundance following enemy attacks, possibly due to associated fitness costs [117], antagonistic interactions with other symbionts [118], and increased susceptibility to alternative natural enemies [119].

### 5.3. Knowledge Gaps and System Advantages

Understanding infection dynamics of facultative symbionts in natural populations remains incomplete. For example, little is known about how rates of horizontal transfer, vertical transmission failure, or other non-selective processes contribute to symbiont population dynamics in the field. Similarly, we lack knowledge of how complex symbiont-mediated phenotypic landscapes—arising from factors such as strain variation and co-infection (see below)—influence field prevalence patterns. Despite these knowledge gaps, information about aphid symbiont prevalence and ecological dynamics under field conditions surpasses that available for other host–symbiont systems. When integrated with experimental and mechanistic understanding of defensive partnerships and extensive aphid community ecology research, this system provides unprecedented opportunities for comprehensive study of symbiont-mediated interactions spanning multiple trophic levels.

## 6. Mechanisms Underlying Symbiont-Mediated Phenotypes

### 6.1. Genomic Foundations and Mobile Elements

Genomic approaches have greatly improved our understanding of aphid facultative symbiont evolution and function [120]. Complete genome sequences are now available for nearly all common aphid facultative symbionts, including *Serratia* [121,122], *Hamiltonella* (pea aphids and *Ceratovacuna japonica*) [123,124,125,126], *Fukatsuia* [85,127,128], *Regiella* (pea aphids and *Myzus persicae*) [129,130], *Rickettsiella* [131], *Arsenophonus* sp. (*Aphis craccivora*: NCBI BioProject PRJNA1231049), and *Wolbachia* (*Pentalonia* aphids: NCBI BioProject PRJEB24287). The only aphid symbiont without an existing genome is *Rickettsia*, although numerous strains of this bacterium isolated from other arthropods have been sequenced [132]. The genomes of facultative symbionts tend to be intermediate in size between the obligate symbiont *Buchnera* and free-living relatives and contain copious mobile elements that influence genome architecture and are often involved in symbiont function [11].

### 6.2. Advances in Cultivation and Sequencing Technologies

Heritable symbionts traditionally resisted laboratory cultivation due to genome erosion and dependence on host-derived factors [133,134]. Early aphid symbiont genome assemblies using short-read sequencing technologies provided limited resolution of mobile DNA elements—critical drivers of phenotypic variation [121,129]. The breakthrough in culturing facultative symbionts independently of their aphid hosts enabled production of contamination-free DNA templates and facilitated long-read genome sequencing capable of capturing the complete structure and content of mobile DNA elements that were previously fragmented or misassembled [124,126,127,128]. Successful axenic cultivation of *Serratia* and *Fukatsuia* isolates [128,135] has opened new avenues for experimental manipulation, allowing researchers to modify symbionts and reintroduce them into aphids for detailed studies of host-microbe interactions [136].

### 6.3. Molecular Mechanisms of Defense

A combination of genomics and experimental biology has been leveraged to understand mechanisms of symbiont defense, with *Hamiltonella* serving as the best-characterized example. The first *Hamiltonella* genome revealed a dynamic architecture encoding copious toxins and pathogenicity factors [123,137], including a double-stranded DNA bacteriophage named APSE. The mosaic genome of APSE contains DNA metabolism and virion assembly regions as well as a virulence module encoding three eukaryotic toxin homologs implicated in defense: cytolethal distending toxin (*cdt*), shiga-like toxins, and YD-repeat proteins [137,138,139,140,141,142,143]. Subsequent studies combining in vivo experiments and genomics isolated phage virulence factors in aphid protection by holding host and bacterial genotypes constant while varying APSE phage presence or type [126,144,145]. In vitro cultivation of *Hamiltonella* with insect cells revealed that, even without aphids present, symbionts produced soluble factors that entered wasp tissues and disabled development [146]. This combined genomic and experimental evidence conclusively demonstrates that phage-derived products kill wasps without requiring aphid-mediated processes and highlights the importance of phage-mediated horizontal gene transfer in conferring anti-parasitoid defense.

### 6.4. Broader Defensive Strategies and Knowledge Gaps

Defensive strategies employed by facultative symbionts broadly fall into three categories: direct interference through toxins [140], resource competition between symbionts and natural enemies [147], and host immune modulation [148]. While symbiont-mediated protection against aphid natural enemies is well-documented, most underlying mechanisms remain unresolved beyond *Hamiltonella*’s anti-parasitoid defenses. However, comparative insights from other systems provide valuable substrate for hypothesis generation and reveal intriguing mechanistic convergence across distantly related host-symbiont partnerships.

In *Drosophila*, for example, *Spiroplasma* confers protection through ribosome-inactivating proteins (RIPs) that target parasitoids and nematodes [149,150]. RIPs are widely occurring *N*-glycosidases that irreversibly inactivate ribosomes by modifying eukaryotic rRNA, and likely function similarly in aphids. This symbiont also protects against pathogens by modulating iron sequestration and enhancing melanization [151].

*Hamiltonella* protection against parasitoids depends on bacteriophage-encoded toxins. These include cytolethal distending toxin subunits (CdtB) that cause DNA damage and cell cycle arrest, and AIP56 toxins that induce apoptosis through NF-κB pathway disruption—mechanisms well-characterized in bacterial pathogens of various eukaryotes [152,153,154].

The defensive role of these toxins gains independent support from their horizontal transfer into the *Drosophila ananassae* genome, where they function as acquired defenses against parasitoids despite their likely origin from facultative symbionts [155,156]. Notably, both *Hamiltonella*/APSE-encoded toxins in aphids and the acquired toxins in *Drosophila* lack certain subunits or domains typical in free-living pathogens, suggesting that their pairing may facilitate cell entry. The apparent targeting of extraembryonic serosa tissues by *Drosophila* toxins [155] suggests that extraembryonic tissues in aphid parasitoids may also represent primary targets.

These findings indicate that defensive symbionts have co-opted toxicity mechanisms originally evolved in free-living bacteria. Critical questions remain, however, regarding the precise mechanisms of toxin delivery, the specific tissues and organs targeted in aphid parasitoids, how aphid hosts avoid self-harm from these defensive products, and the coevolutionary dynamics between aphid immune systems and symbiont-mediated defenses.

Knowledge of other protective mechanisms in aphids remains limited compared to toxin-mediated defenses. Symbiont-mediated immune priming represents one potential mechanism by which bacterial partners can upregulate host antimicrobial peptides in anticipation of an enemy attack. However, despite evidence for this phenomenon in other systems [157,158] and the known effects of aphid facultative symbionts on innate immunity components [159,160,161] no confirmed cases of immune priming have been reported in aphids. Even pathogen exposure fails to prime aphid immunity [162].

Resource competition offers another potential protective avenue, where facultative symbionts directly compete with natural enemies for essential nutrients or metabolites. Examples include *Wolbachia* competing with *Drosophila* C virus for cholesterol [163] and *Spiroplasma* competing with parasitoids for lipids [147]. Yet no analogous competitive interactions have been identified in aphid systems. However, given the metabolic intimacy between aphids and their obligate symbionts, even modest competitive effects may prove decisive in determining infection outcomes. Yet, the dynamic nature of these interactions remains poorly understood. Specialized natural enemies may themselves alter aphid nutritional physiology during attack, potentially affecting symbiont titer, toxin biosynthesis, host immune activation, or the competitive landscape between symbionts and enemies. These cascading effects could either enhance or compromise defensive efficacy, highlighting the need for integrative approaches that consider the full complexity of tripartite host-symbiont-enemy interactions.

In contrast to defensive scenarios, the mechanistic basis for transitioning to co-obligate symbiosis is straightforward based on genomic inference, wherein co-obligate symbionts typically develop from facultative ones after *Buchnera* loses key metabolic functions that the facultative symbiont retains [27].

This mechanistic gap limits our understanding of why symbiont-mediated defense can fail under various conditions, including elevated temperatures [71], increased parasitoid pressure through superparasitism [164], or novel parasitoid encounters [165]. Progress is further hampered by research focusing primarily on ultimate outcomes (e.g., mummification, fungal growth) rather than examining the intermediate processes during parasitoid development or pathogen infection that determine defensive success.

Indeed, a fundamental limitation constraining mechanistic understanding is the narrow focus on host-symbiont perspectives without adequate consideration of natural enemy biology. Comprehensive understanding of symbiont-mediated defenses requires detailed knowledge of parasitoids and pathogens, cellular targets, and developmental vulnerabilities. This enemy-centered perspective becomes particularly critical given that symbiont-mediated benefits often conflict directly with agricultural management objectives, necessitating research approaches that integrate natural enemy biology with host-symbiont interactions to develop more effective and sustainable pest control strategies.

## 7. Strain Variation, Co-Infections, and Interactions with Endogenous Host Traits Create Complex Phenotypic Landscapes

The field has evolved beyond characterizing single symbiont species in isolation to examine strain-level variation, interactive effects of co-occurring facultative symbionts, and host-symbiont integration.

### 7.1. Strain-Level Variation Drives Phenotypic Diversity

The anti-parasitoid symbiont *Hamiltonella* exemplifies the importance of strain-level variation, with APSE phages driving substantial heterogeneity in parasitism resistance. In North American pea aphid populations, all examined *Hamiltonella* strains carrying APSE confer some protection against their primary co-evolved enemy *Aphidius ervi*, though efficacy varies with specific APSE variant [39,126]. This protective specificity extends across aphid species, with different *Hamiltonella* isolates targeting distinct wasp species or even specific genotypes within species [45,165,166,167,168,169,170,171]. Such strain-specific protection can mediate competition between rival parasitoid species and influence community dynamics, potentially affecting biological control efficacy [172,173,174,175]. Some *Hamiltonella* isolates provide no protection, due to phage loss [144,176], potential specificity mismatches with tested parasitoids [47], or transition to co-obligate symbiosis or other non-defensive roles [138].

Strain variation patterns differ among symbiont species. *Regiella*-mediated protection against specialized fungal pathogens like *Pandora neoaphidis* shows limited strain-level variability [93], though strains can establish at different titers affecting host immune responses and fitness [177]. In contrast, *F. symbiotica* strains exhibit dramatic functional variation—some confer protection against parasitoids, fungi, and heat stress, while others provide none of these benefits [48,118]. Strain identity often explains phenotypic variation better than species identity [178], highlighting the critical importance of characterizing symbiont diversity at the strain level.

### 7.2. Co-Infection Dynamics and Symbiont Interactions

Multiple infections with 2–4 facultative symbionts are common in aphids, with most evidence from pea aphid studies [115,179]. In natural populations, certain symbiont combinations occur more or less frequently than expected by chance [180], indicating non-random assembly of heritable symbiont communities. These patterns extend to strain-level associations, such as a common *Fukatsuia* strain that almost invariably associates with B-clade *Hamiltonella* in alfalfa biotype pea aphids [118,126].

Co-infection patterns likely emerge through multiple mechanisms: selection on protective phenotypes at the host level [41,181,182,183], direct symbiont interactions through competition or cooperation [127,184], indirect associations through hitchhiking effects [118,185], and context-dependent co-transmission efficiency [180]. The functional consequences of co-infection are complex and variable—protective phenotypes may be maintained [41], lost [186], or show variable outcomes [181]. Co-infection can sometimes ameliorate fitness costs imposed by individual facultative symbionts [118,181,187].

### 7.3. Integration with Endogenous Aphid Defenses

Early recognition of clonal variation in pea aphid resistance to parasitoids [188,189] was partially overshadowed by discoveries of defensive symbiosis, leading to assumptions that aphids largely outsource protection to facultative symbionts. However, pea aphids exhibit substantial endogenous resistance to both parasitoids and fungal pathogens [71,165,190]. Notably, pea aphid biotypes that more frequently harbor anti-fungal symbionts show higher rates of endogenous resistance, yet within biotypes, no correlation exists between endogenous resistance and symbiont presence [191], suggesting coordinated protection under strong selection pressure.

Understanding endogenous defenses is crucial for appreciating host–symbiont partnership evolution. Some aphid species have acquired *cdtB* toxin genes through horizontal transfer [192], but it remains unclear whether these contribute to endogenous resistance in aphids, and if so, whether they function independently or in coordination with factors traditionally involved in thwarting parasitoid development, including immune cells. While *Drosophila* offers superior genetic tools, aphids provide unique evolutionary insights through experimental flexibility and their natural associations with diverse defensive symbionts. This system thus offers exceptional opportunities to study the origins and ongoing evolution of defensive partnerships, whether mediated by symbiont-encoded toxins or canonical cellular and humoral immunity.

### 7.4. Future Directions in Symbiont Complexity

The transition from single-symbiont studies to examining strain variation, co-infections, and host integration clearly reveals that mechanistic understanding lags behind descriptive knowledge of these complex phenotypic landscapes. Current aphid gene knockout methods have largely proven slow and inconsistent, limiting investigation of symbiont-mediated and endogenous defense interactions in vivo. CRISPR requires months due to sexual reproduction requirements [193], traditional RNAi shows weak knockdown in aphids [194]. Alternatives have been attempted, such as engineering bacteria to continuously produce gene-silencing dsRNA inside aphid guts, but these also failed to consistently achieve reliable gene knockdown [195]. Developing improved genetic tools would leverage the aphid–symbiont system’s experimental tractability to dissect how multiple defensive layers coordinate in natural populations. Technological advancements could transform our ability to predict ecological outcomes and understand the evolutionary drivers of protective partnerships. Enhanced genetic approaches would enable precise characterization of both host and symbiont defensive mechanisms, providing critical insights into system vulnerabilities under environmental stressors such as thermal stress or novel natural enemies, thereby informing more targeted and effective biological control strategies.

## 8. Aphid Symbiont Systems: Cascading Effects, Coevolution, and Agricultural Applications

### 8.1. Ecological Network Effects

Symbiotic relationships extend beyond direct host–symbiont interactions to influence broader ecological networks [196]. For example, when symbiont-mediated defenses reduce aphid parasitism rates, yielding fewer mummified individuals, hyperparasitoid populations suffer from resource limitation [197]. The effectiveness of defensive services provided by facultative symbionts varies with ecological situation—diminishing in ant-tended aphids and fluctuating based on infection patterns in neighboring aphid populations [115,175,198]. Paralleling nutritional symbioses, where hosts become metabolically dependent on microbes, aphids harboring defensive symbionts often exhibit reduced defensive behaviors [199], suggesting evolutionary trade-offs in defensive strategies. This ecological complexity demonstrates how symbiotic relationships simultaneously respond to and reshape their surrounding environment, creating cascading effects throughout multi-trophic networks.

### 8.2. Coevolutionary Arms Races

The interplay between hosts, facultative symbionts, and natural enemies extends beyond immediate ecological consequences to shape evolutionary trajectories through co-evolutionary arms races operating across multiple scales. Parasitoid wasps have evolved sophisticated counter-adaptations to symbiont defenses—including sensory mechanisms to detect and avoid symbiont-protected hosts [200]—and both physiological and behavioral strategies, such as strategic superparasitism, to overcome symbiont protection [164,170,199,201].

This reciprocal selection operates at the genotypic level, with symbionts selecting for specific wasp genotypes and vice versa [202]. Parasitoid specificity may drive symbiont strain diversity [203] and with parasitoids pre-adapted to particular *Hamiltonella* strains achieve higher parasitism success than non-adapted counterparts [204]. In turn, parasitoid genetics reciprocally shape symbiont diversity ([205,206], but see [207]). These interactions potentially influence parasitoid host range [208] and drive parasitoid diversification [209].

However, evolutionary patterns show considerable geographical variation. Limited evidence for co-adaptation exists in black bean aphids [210] and pea aphids [171], suggesting that local ecological conditions may constrain or facilitate co-evolutionary dynamics. Specialized microbial pathogens, particularly fast-reproducing viruses, likely exhibit even more pronounced evolutionary and biogeographical patterns, though these dynamics remain critically understudied.

### 8.3. Maintaining Symbiont Diversity

The aforementioned co-evolutionary dynamics raise fundamental questions about the maintenance of phenotypic diversity within symbiont communities. A particularly intriguing puzzle concerns the persistence of symbiont strains that provide only intermediate levels of protection against natural enemies [40,211]. If natural selection favored maximum defensive efficacy, these moderately protective variants should be outcompeted by highly effective defensive strains—particularly those with minimal fitness costs [75]—yet they continue to persist in aphid populations.

This evolutionary paradox suggests that simple defensive efficacy alone cannot explain symbiont community structure. Several mechanisms may maintain this diversity: trade-offs between different protective services where no single strain provides optimal defense against all enemies, context-dependent benefits that favor different strains under varying ecological conditions, or complex frequency-dependent selection pressures where rare strains gain advantages through enemy adaptation lag. Additionally, spatial and temporal heterogeneity in selection pressures may create refugia for less effective strains, while stochastic processes during symbiont transmission could maintain suboptimal variants despite directional selection.

### 8.4. Agricultural Management Implications

This complex web of co-evolutionary relationships has significant implications for agricultural pest management. Facultative symbionts that protect against biological control agents present particular challenges for managing both established crop pests [40] and invasive species [212]. The effectiveness of parasitoid wasps deployed as biocontrol agents fundamentally depends on their ability to overcome both endogenous and symbiont-mediated defenses in target pest populations.

These defensive symbionts create powerful selection pressures and generate eco-evolutionary feedback loops where ecological and evolutionary processes become intertwined, potentially undermining parasitoid-mediated biological control strategies [78,96,172]. Variation in resistance levels, defense costs, and dispersal patterns may promote long-term persistence of both evolutionary and ecological diversity [96,213], complicating predictions about biocontrol efficacy.

However, facultative symbionts also offer novel management opportunities. Beyond parasitoid resistance, symbionts providing antiviral protection hold particular promise given aphids’ role as major vectors of economically damaging plant viruses. Emerging management approaches could leverage protective *Regiella* strains that reduce virus transmission through direct transfection techniques [214] or *Pandora* fungal applications. The latter strategy offers dual benefits: reducing aphid populations through selective mortality while simultaneously promoting the spread of protective symbiont strains among surviving aphids, ultimately decreasing plant virus transmission rates.

These applied interventions represent a promising frontier where fundamental understanding of symbiont ecology translates into practical pest management solutions, transforming potential obstacles into management tools.

## 9. Conclusions

Facultative symbionts function as ecological mercenaries—microbial partners that offer protective services against natural enemies in exchange for shelter and nutrition yet maintain the flexibility to switch between host lineages and species, effectively spreading their defensive capabilities across aphid communities.

**Figure 2:**
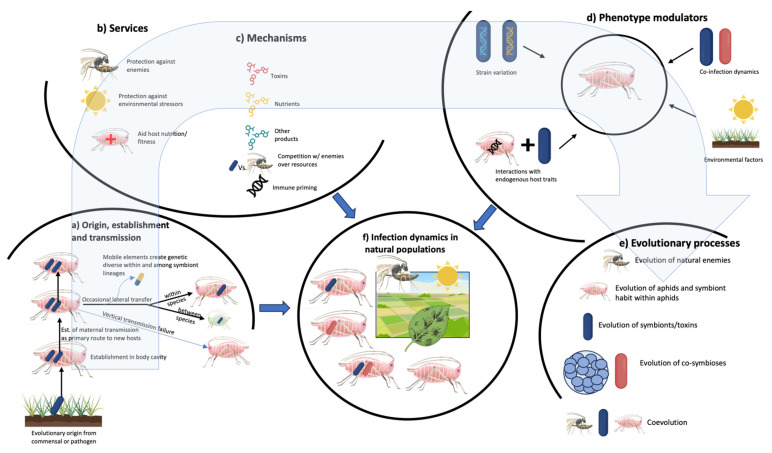
Integrative framework for understanding facultative symbioses in aphids. Understanding facultative symbioses in aphids requires examining multiple interconnected components: (**a**) evolutionary origins and transmission mechanisms, (**b**) services provided by symbionts, (**c**) mechanisms underlying symbiont activity, (**d**) multipartite arrangements creating modular and shifting symbiosis phenotypes, and (**e**) coevolution of hosts, symbionts, and interacting organisms. These components collectively shape (**f**) infection dynamics in natural populations and their ecological consequences. Integrating these perspectives is essential for advancing both fundamental understanding of symbiosis evolution and practical applications in agriculture and invasive species management. Created in BioRender. Kucuk, R.A. (2025). https://BioRender.com/v3ae13b.

The integrative framework presented in Figure 2 captures why aphid–facultative symbiont systems have become powerful models for understanding ecological symbioses. Rather than functioning as isolated components, these relationships require examination of interconnected processes—from symbiont establishment and protective mechanisms to population dynamics and evolutionary feedbacks. The cyclical interactions among these components—where outcomes at one level influence processes at others—exemplify the systemic complexity that makes these partnerships exceptionally valuable for revealing fundamental principles of symbiosis and ecology.

While improvements in the gene knockdown and knockout are still needed, the ability to create experimental lines controlling for host and symbiont contributions represents a key advantage positioning this system at the forefront of symbiosis research. However, this manipulative power remains underutilized for addressing fundamental questions in symbiont-mediated multi-trophic interactions. In anti-parasitoid symbiosis, for example, there is little integration of symbiont biology with parasitoid developmental strategies, limiting our understanding of how aphid and symbiont factors work together to thwart parasitoids. Similar gaps exist in aphid–pathogen interactions, where the mechanistic details of protection remain largely unresolved.

The field has evolved from identifying and studying individual symbiont phenotypes in isolation to increasingly embracing the fuller ecological context. This shift encompasses abiotic factors like temperature, co-infections with multiple symbiont species, interactions with plant-associated microbes, and the broader community of natural enemies and mutualists that collectively shape food web interactions in nature. This holistic approach promises to reveal how symbiotic partnerships function as integrated components of complex ecological networks rather than as simple pairwise associations.

## Data Availability

No new data were created or analyzed in this study. Data sharing is not applicable to this article.
